# Application of nucleophilic substitution reaction for sensitive determination of heptaminol hydrochloride in pharmaceuticals

**DOI:** 10.1186/s13065-024-01327-8

**Published:** 2024-11-07

**Authors:** Mahmoud Abdelgaleel, Dalia M. Nagi, Mohamed Oraby, Sayed M. Derayea, Pakinaz Y. Khashaba

**Affiliations:** 1 Pharmaceutical Chemistry Department, Faculty of Pharmacy, Deraya University, New Mina City, Minia Egypt; 2 Pharmaceutical analytical Chemistry Department, Faculty of Pharmacy, Minia University, Minia, Egypt; 3 Pharmaceutical analytical Chemistry Department, Faculty of Pharmacy, Sohag University, Sohag, Egypt; 4 Pharmaceutical Chemistry Department, Faculty of Pharmacy, Sphinx University, New Assiut City, Assiut Egypt; 5 Pharmaceutical analytical Chemistry Department, Faculty of Pharmacy, Assiut University, Assiut, Egypt

**Keywords:** HTM-HCl, Spectrofluorimetry, Derivatization, Dansyl chloride&Method validation

## Abstract

A straightforward and sensitive spectrofluorimetric approach was established for the determination of heptaminol hydrochloride (HTM-HCl) based on the derivatization of the drug through its reaction with 5-dimethylaminonaphthalene-1-sulfonyl chloride (Dansyl chloride). The reagent underwent a nucleophilic substitution of its chlorine atom with HTM to give N-(5-dimethylaminonaphthalene-1-sulfonyl)-6-amino-2-methylheptan-2-ol. The highly luminescent derivative was extracted using methylene chloride and subjected to analysis at an excitation wavelength of 345 nm and an emission wavelength of 490 nm. The chemical reaction occurred within an aqueous environment buffered with a 0.1 M borate buffer solution adjusted to pH 10.5. Experimental findings indicate that the proposed method displays sensitivity and linearity across a concentration range from 0.03 to 2 µg mL^− 1^. The method achieves lower detection and quantification limits of 0.016 and 0.048 µg mL^− 1^, respectively. The analytical validation of this method followed the guidelines outlined by the International Council of Harmonization (ICH). This approach was applied effectively for quantifying the medication in both tablet and oral drops formulations available on the market, demonstrating excellent recovery of 98.95 ± 0.45 for tablets and 99.37 ± 0.24 for oral drops with no interference from excipients.

**Supplementary Information:**

The online version contains supplementary material available at 10.1186/s13065-024-01327-8.

## Graphical abstract

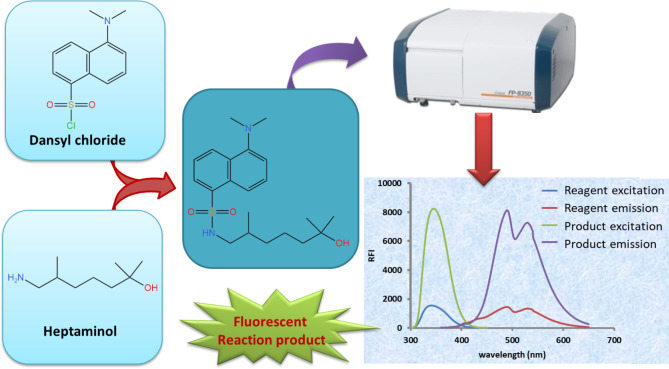

## Introduction

Heptaminol hydrochloride is 6-amino-2-methylheptane-2-ol hydrochloride. It exerts a positive inotropic effect on the cardiovascular system resulting in increasing the coronary blood flow and slight peripheral vasoconstriction which makes the drug suitable for the management of orthostatic hypotension. The drug is also used for catecholamine weaning in septic shock [1–4].

Officially, HTM-HCl can be determined by a potentiometric titration method as mentioned in European pharmacopoeia [5]. On the other hand, various analytical techniques were reported for determination of the cited drug. Chromatographic methods such as HPLC with UV detection [6], HPLC fluorescence detection [7, 8], HPLC MS detection [9–12] and GC [13–15] often require thorough clean-up procedures. These processes can be labor-intensive and time-consuming, particularly when a mass spectrometer detector is utilized. Additionally, the instrumentation involved in these techniques is expensive, further contributing to the overall cost of the analysis process. The reported TLC [16] method comprised the initial step of extracting the sample with ether under alkaline conditions, followed by the subsequent reaction of the extracted HTM-HCl with 4-chloro-7-nitrobenzo-2,1,3-oxadiazole prior to the separation process.

The spectrophotometric techniques are always characterized by its simplicity [17]. However, spectrophotometric methods for analysis of HTM-HCl [18, 19] lack of sufficient sensitivity for determination of the studied drug. The reported conductometric method for analysis of HTM-HCl [20] is considered as non- selective technique as it could be affected by presence of contaminated ions as shown in supplementary Table 1. By looking to reported spectrofluorimetric methods for analysis of HTM-HCl [18, 21–25], these methods depend on using expensive reagents, elevated temperature or have a relative low sensitivity.

The main advantage of spectrofluorimetric techniques is its high sensitivity beside to the simplicity as cleared from many previous reported works [26]. The presence of amine function group in the structure of the drug allows the derivatization with dansyl chloride (DNS-Cl) yielding a highly fluorescent product [27–32]. This work presents a new spectrofluorimetric method for determination of the cited drug based on dansylation reaction. The proposed method is characterized by its simplicity, rapidity, sensitivity, cost-effectiveness, and compatibility with ambient temperature conditions. It is particularly well-suited for the analysis of the drug within tablet formulations and oral drops.

## Experimental

### Instrument

The spectrofluorimetric measurements were conducted using a Jasco FP-8350 spectrofluorimeter. The equipment was equipped with a 150 W Xenon-arc lamp and operated at a photomultiplier tube (PMT) voltage of 400 V. The emission and excitation monochromators were configured with a slit width of 5 nm, while a scan rate of 1000 nm.min^− 1^ was selected for the measurement.

### Materials and chemicals

DNS-Cl was procured from Sigma (St. Louis, USA). It was prepared by dissolving 20 mg in 100 mL of acetone giving a solution with a concentration of 0.02% w/v. Sodium bicarbonate, sodium hydroxide, boric acid, methylene chloride, methanol, isopropanol, ethyl alcohol, and 1-butanol were obtained from El Nasr Company for Chemicals (Cairo, Egypt). HTM-HCl was generously provided as a gift by PHARCO Company for Pharmaceuticals Industries (Cairo, Egypt). Corasore^®^ tablets containing 150 mg of the drug per tablet and Corasore oral drops with a concentration of 150 mg mL^− 1^ of the drug were obtained from a local pharmacy in Egypt. Borate buffer preparation was carried out by mixing different amounts of NaOH and boric acid all in concentration of 0.1 M to obtain the target pH.

### Standard drug solution

To get the stock solution of HTM-HCl, an exact quantity of 10.0 mg of the pure drug was carefully placed into a 100 mL volumetric flask. Subsequently, it was dissolved thoroughly in distilled water and mixed until completely uniform. The volume was then adjusted to the marked line using the same solvent. Following this step, portions of the prepared stock solution were diluted further to reach a final volume of 100 mL, achieving the desired concentration for subsequent use.

### Procedure for construction of the calibration graph

Different concentrations of the drug solution ranging from 0.03 to 2.0 µg mL^− 1^ were introduced into 10 mL glass tubes fitted with stoppers. Following this, 0.4 mL of borate buffer solution with a pH of 10.5 and 0.6 mL of the prepared DNS-CL solution were added to each tube. For thirty minutes, the tube was kept at room temperature. Following excitation at 345 nm, the fluorescent product was extracted using 3 mL methylene chloride for 3 times, and the fluorescence was measured at emission wavelength 490 nm. The test experiment was conducted beside a blank.

### Procedure for tablets formulation

Twenty tablets of the commercial Corasore^®^ product were accurately weighed, ground, and rendered uniform. An amount corresponding to 10.0 mg of the medication was subsequently subjected to ultrasonic treatment with distilled water in a standard flask of 100 mL for duration of 20 min. Post-sonication, the solution underwent dilution to reach the mark, followed by filtration. The resulting filtrate was subsequently diluted to obtain solutions falling within the requisite concentration range, and the stipulated analytical methodologies were implemented.

### Procedure for oral drops

An aliquot of oral solution was diluted with water to the required working drug solution. Different volumes of the resulting working solution covering the linear range were transferred to stoppered glass test tubes and general analytical procedures were applied.

## Results and discussion

**Fig. 1:**
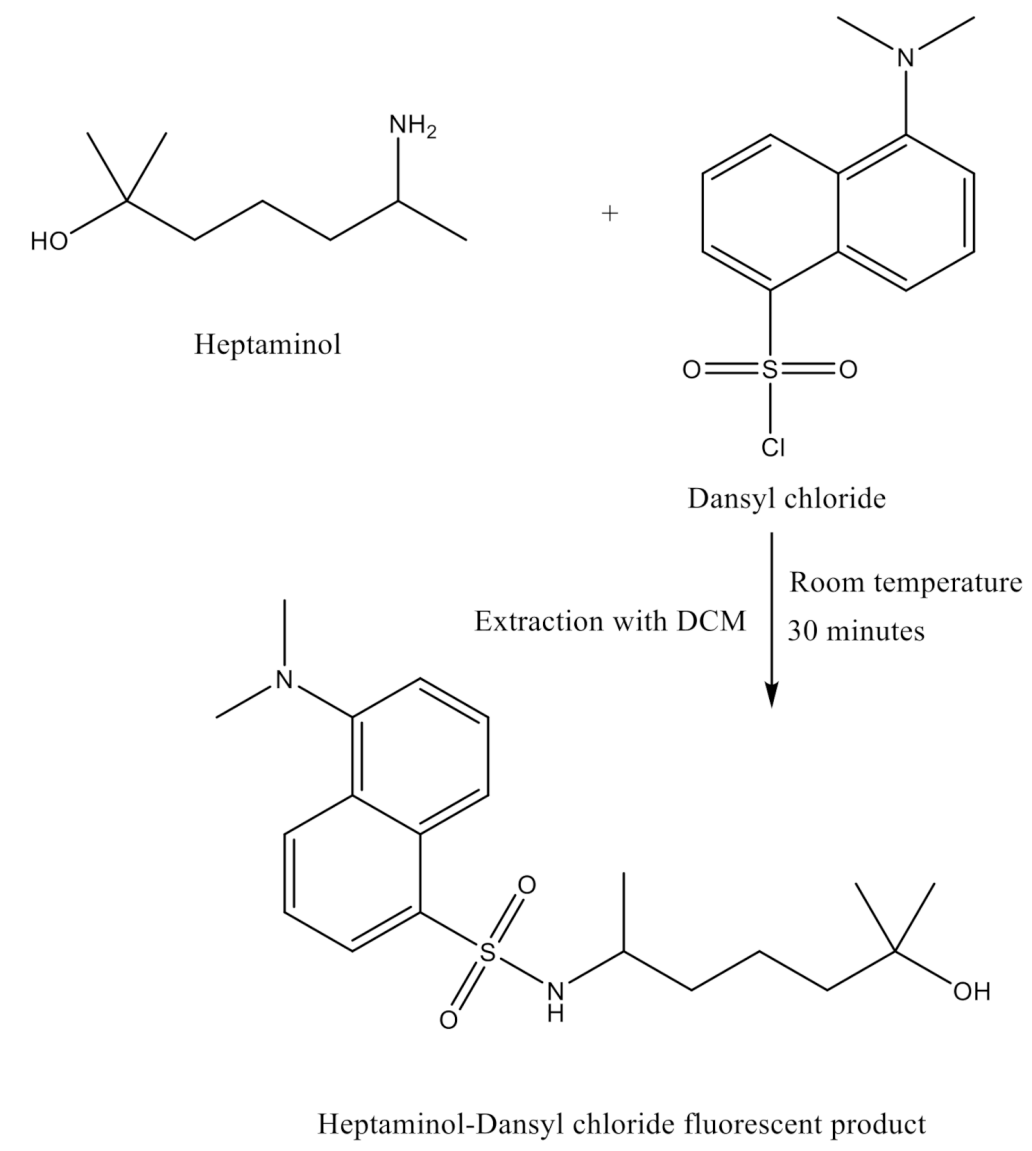
The suggested reaction pathway of HTM-HCl and DNS-Cl

**Fig. 2:**
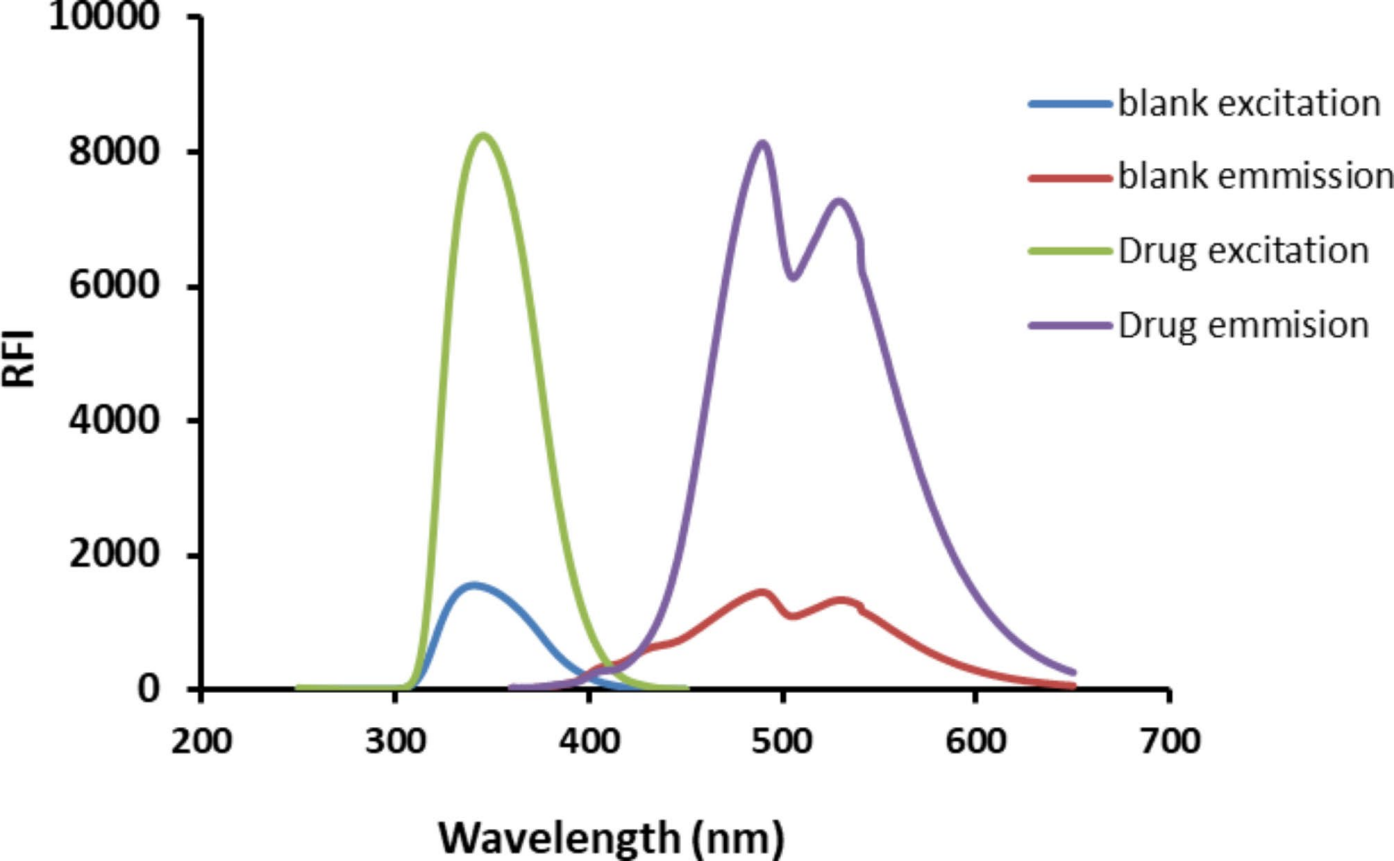
Fluorescence spectra of 2 µg mL^− 1^ HTM-HCl – DNS-Cl reaction product and blank containing DNS-Cl (0.02% w/v)

The presence of the primary amino group in the inherent aliphatic structure of HTM-HCl facilitates its derivatization with DNS-Cl, resulting in the generation of a fluorescent derivative, as illustrated in Fig. 1. Interaction with DNS-Cl initiates the conversion of the drug into a highly luminescent product. Following this reaction, the resultant fluorescent species is extracted into methylene chloride and its emission intensity is recorded at 490 nm after subsequent excitation at 345 nm with a stock shift of 145 nm (Fig. 2). It should be noted that, the excitation and emission wavelengths for both DNS-Cl and its product with HTM are relatively similar. However, their fluorescence intensities were completely different. The similarity in excitation and emission wavelengths arises from the structural integrity of the dansyl group, which retains its fluorophore characteristics in both forms. However, the fluorescence intensities differ significantly due to the removal of the chloride atom attached to the sulfonyl group in the reagent and its replacement with amino derivative. The presence of the chloride atom in the reagent greatly quench its fluorescence due to high electronegativity of the chloride that can withdraw electron density from the naphthalene moiety This can destabilize the excited state and reduce the fluorescence efficiency. In addition, chlorine is a heavy atom which can lead to increased non-radiative pathways for energy loss, such as vibrational relaxation, which decreases the amount of energy emitted as fluorescence. Furthermore, Chlorine atom can facilitate intersystem crossing to triplet states, where the molecule can lose energy through non-radiative processes instead of returning to the ground state via fluorescence.

### Selection of optimum experimental parameters

A comprehensive investigation was conducted to assess the influence of various parameters such as pH, DNS-Cl volume, buffer volume, reaction duration, and choice of extracting solvent on the interaction between HTM-HCl and DNS-Cl. This inquiry aimed to identify the most suitable reaction conditions.

#### Influence of pH

**Fig. 3:**
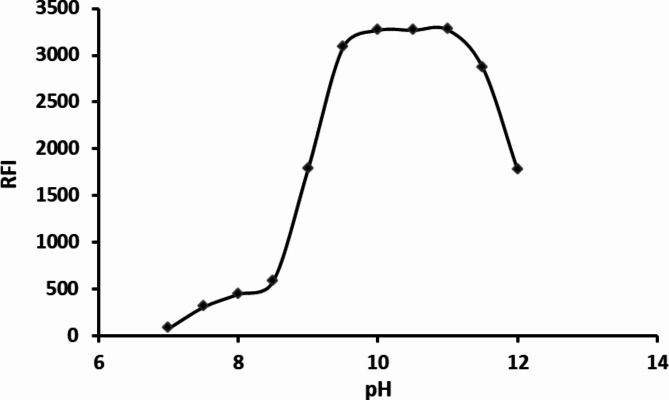
Effect of pH of borate buffer on the observed RFI for the reaction product of 2 µg mL^− 1^ HTM-HCl and DNS-Cl

The recommended procedure was performed using varied pH of the buffer solution in the range of 7.0–12.0 using 0.1 M borate buffer. The product fluorescence is directly proportional to pH elevation until reaching pH 9.5 and remains nearly constant till pH 11. The fluorescence intensity exhibits a notable decrease with a further rise in pH. So, pH of 10.5 was selected as the most suitable pH for this experiment (Fig. 3).

#### Influence of buffer volume

**Fig. 4:**
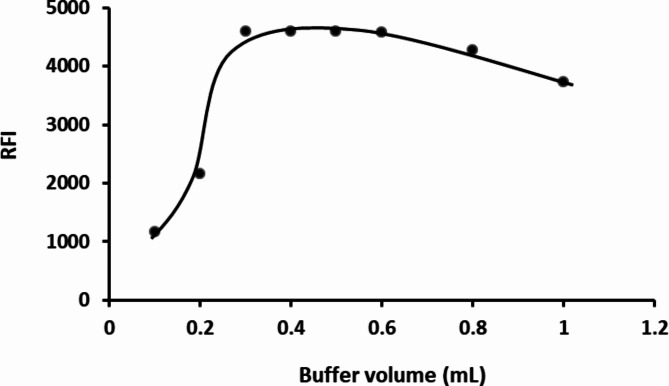
Impact of borate buffer (pH 10.5) volume on the reaction of 2 µg mL^− 1^ HTM-HCl with DNS-Cl

To assess the impact of 0.1 M borate buffer volume on the fluorescence of reaction product, different aliquots ranging from 0.1 to 1.0 mL were examined. The findings revealed a rise in fluorescence intensity with increasing buffer volume up to 0.3 mL, after which the intensity plateaued until reaching 0.6 mL. Beyond this point, further volume increase led to a decline in fluorescence intensity. Consequently, 0.4 mL was identified as the most suitable volume for this experiment. (Fig. 4)

#### Influence of time

**Fig. 5:**
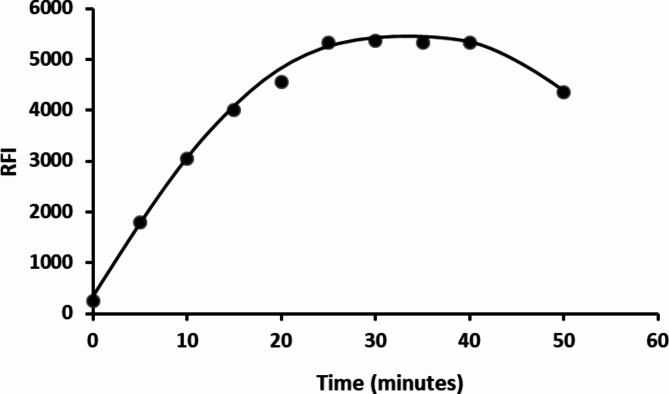
The impact of time reaction on the RFI of the product between 2 µg mL^− 1^ HTM-HCl and DNS-Cl

The fluorescence intensity of the resulting product was measured immediately and at different time intervals. (5–50 min.). Results showed that the reaction between the cited drug and dansyl chloride is highly dependent on time and were completed after 25 min. The product exhibited stability for an additional 15 min. Nonetheless, prolonged standing led to a significant inhibition of fluorescence, so 30 min was chosen as the most suitable time for the reaction. (Fig. 5).

#### Influence of DNS-Cl volume

**Fig. 6:**
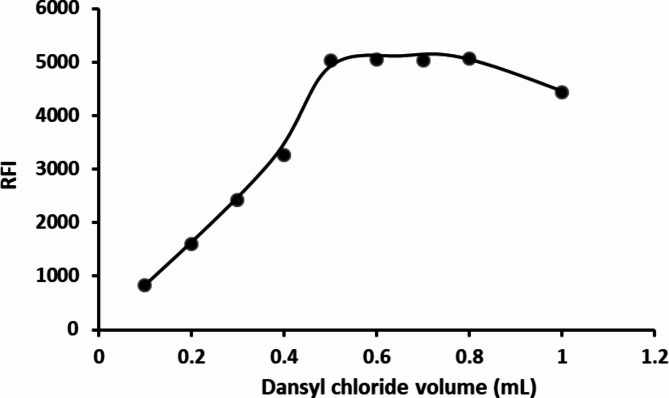
The impact of varying volumes of DNS-Cl on the RFI of its reaction product with 2 µg mL^− 1^ HTM-HCl

A range of volumes of dansyl chloride, spanning from 0.1 mL to 1 mL, was scrutinized to evaluate their impact on the fluorescence intensity of the resultant product. Observations revealed a progressive enhancement in fluorescence as the volume of the reagent was incremented until reaching 0.5 mL. Subsequently, the fluorescence intensity remained relatively stable up to 0.8 mL. Nevertheless, a discernible decline in fluorescence intensity was noted upon the addition of higher volumes of the reagent. Consequently, 0.6 mL was identified as the most suitable volume for this experiment. (Fig. 6).

#### Influence of solvent

The fluorescence intensity of reagent blank in water was very high, therefore different solvent were tried to reduce the reagent blank reading. Thus, various water-miscible solvents were employed for dilution of the fluorescent product without extraction. The examined solvents were methyl alcohol, ethyl alcohol, isopropanol, and 1-butanol. However all these solvents also demonstrated elevated blank values and thus, were consequently eliminated from consideration. To address this issue, the product was subjected to extraction using methylene chloride which was chosen because of its higher extraction efficiency and its relatively lower toxicity compared to other organic solvents. In addition, the fluorescence intensity of the blank was relatively low as seen in (Fig. 2).

### Validation of the suggested approach

The proposed method was validated following the ICH guiding rules [33] regarding the linearity, range, detection and quantitation limits, robustness, accuracy and precision.

#### Linearity and range

The provided analytical strategy was utilized to evaluate multiple drug solutions at different concentration levels. Through the plotting of relative fluorescence intensity at 490 nm against drug concentration in µg.mL^-1^, a calibration graph was established for the spectrofluorimetric approach. Notably, the proposed method displayed excellent linearity within the concentration range of 0.03 to 2.0 µg.mL^-1^. The data tabulated in Table 1 corroborate the high degree of linearity achieved with this approach.

**Table 1:** Analytical parameters for analysis of cited drug with the suggested method

Parameter	Value
linear range (µg mL^− 1^) slope intercept standard deviation of slope standard deviation of residuals standard deviation of intercept determination coefficient (r^2^) correlation coefficient (r) LOQ (µg mL^− 1^) LOD (µg mL^− 1^)	0.03-2.0 3368.4 -16.166 15.543 29.8 16.093 0.9999 0.9999 0.048 0.016

The limit of detection (LOD) and limit of quantitation (LOQ) were determined following the guidelines provided by the International Council for Harmonization (ICH) [33] by applying the equation: LOD = 3.3σ/s and LOQ = 10σ/s whereas σ represent the standard deviation of intercept, while s is the slope of the graph. Value of LOQ was 0.048 µg mL^-1^ while LOD was 0.016 µg mL^-1^.

#### Accuracy

The accuracy of the suggested method was assessed by determining the % recovery of three distinct HTM-HCl concentrations. Each experiment was conducted in triplicate, and the results are presented as the mean percentage recovery along with the standard deviation in Table 2. The close alignment of the % recovery values with 100% across all investigated concentrations provides compelling evidence of the method’s accuracy.

**Table 2:** Accuracy evaluation for the proposed method

Sample number	Concentration level (µg mL^− 1^)	%Recovery* ± SD
1	0.05	97.47 ± 1.02
2	1.00	97.36 ± 0.92
3	2.00	98.7 ± 0.28

*Values are the average of 3 measurements, SD is the standard deviation

#### Precision

In order to assess both intraday and interday precision, three varying concentrations falling within the linearity range were tested using the proposed strategy. Intraday precision was evaluated by conducting experiments on the same day, whereas interday precision was determined by performing experiments over three successive days. All experiments were conducted in triplicate. The high precision of the proposed method was demonstrated by the low % RSD (relative standard deviation) values, as detailed in Table 3.

**Table 3:** Precision and accuracy evaluation of the suggested method at inter – and intra-day levels

Sample number	Concentration level ( µg mL^− 1^)	Intra-day precision		Inter-day precision	
		%Recovery ^a^ ± SD	%RSD	%Recovery ^a^ ± SD	%RSD
1	0.05	97.87 ± 0.34	0.35	98.26 ± 2.08	2.12
2	1.00	97.15 ± 1.08	1.11	97.80 ± 0.59	0.61
3	2.00	98.50 ± 0.87	0.89	98.94 ± 0.34	0.35

^a^ Values are the average of 3 measurements, RSD is the relative standard deviation and SD is standard deviation

#### Robustness

The method’s robustness was evaluated by implementing minor alterations to the experimental parameters including DNS-Cl volume, buffer volume, and pH, then computing the percent recovery. Data listed in Table 4 confirm that there was no significant effect for the small change of these parameters on method performance.

**Table 4:** Evaluation of method robustness

Parameter		%Recovery ^a^ ± SD
pH	10.3 10.5 10.7	100.45 ± 0.47 99.71 ± 0.62 99.87 ± 0.40
Borate buffer volume (mL)	0.3 0.4 0.5	100.29 ± 0.33 99.65 ± 1.01 100.01 ± 0.89
0.02% dansyl chloride volume (mL)	0.5 0.6 0.7	99.21 ± 1.01 100.66 ± 0.07 100.09 ± 0.97

^a^ Values are the average of 3 measurements and SD is the standard deviation

### Application of the proposed method analysis of HTM-HCl pharmaceutical formulations

The spectrofluorimetric method investigated in this study demonstrated efficacy in estimating HTM-HCl in both tablet and oral drop formulations. Notably, high % recovery values of 98.9 ± 0.04 for tablets and 99.37 ± 0.24 for oral drops were attained. A comparative analysis between the proposed method and a previously reported approach [22] conducted through t-value and F-value calculations, indicated no significant deviation from theoretical values at a 95% confidence interval. This underscores the method’s adherence to accepted levels of accuracy and precision. Furthermore, the results presented in Table 5 corroborate the absence of interference from common excipients.

**Table 5:** Application to tablet dosage form and comparison with reported method

Dosage form	Labeled content	Mean %Recovery ^a^± SD		t-value^b^	F-value^b^
		Proposed method	Reported method [25]		
Corasore^®^ tablets	150 mg /tablet	98.95 ± 0.45	100.12 ± 0.56	2.8	1.591
Corasore^®^ oral drops	150 mg.mL^− 1^	99.37 ± 0.24	99.5 ± 0.57	0.386	5.34

^a^ Values are the mean of 3 measurements, SD is the standard deviation

^b^ The tabulated t-value and F‐value at the 95% confidence limit are 2.78 and 6.39, respectively

### Comparison of the proposed method with the published fluorimetric methods

Six spectrofluorimetric methods [18, 21–25] were previously reported for the determination of HTM-HCl. A comparison of the current method with these published spectrofluorimetric methods is presented in supplementary Table 2. As shown in the table, four of these methods [18, 22, 23, 25] had lower sensitivity for HTM-HCl than the proposed spectrofluorimetric method. In addition, no heating step was need compared to the method based on condensation reactions [18, 22, 25]. Furthermore, the utilized reagent (DNS-Cl) is relatively cheap compared to the extremely expensive fluorescamine reagent [23]. Although the reported methods [21, 24] had a comparable analytical performance to the proposed method, the proposed method based on extraction of the fluorescent product to methylene chloride which has relative low toxicity compared to chloroform used in DAS derivatization method [21]. The proposed method also depends on using a single non expensive reagent compared to the orthophthalaldehyde/2ME method [24] with a larger stock shift. The excitation/emission wavelengths were 334/451 (Δλ = 117 nm) in the reported method and 345/490 nm in the proposed method. Thus, the proposed method has a very large Stock shift, (Δλ = 145 nm). Therefore, the proposed method has the merit of the, simplicity, and cost effective.

## Conclusion

A highly sensitive spectrofluorimetric method was developed based on the dansylation of the primary amine of HTM-HCl with DNS-Cl. This method was deemed suitable for analyzing the drug in its pure form, tablet formulations, and oral drops. Notably, the approach is characterized by simplicity, rapidity, sensitivity, and the ability to be performed at ambient temperature without heating. The high sensitivity is evidenced by the low values of LOD (0.016 µg mL^− 1^) and LOQ (0.048 µg.mL^− 1^). As a result, it can be effectively applied for routine analysis of the drug in quality control units. The reported analytical techniques, the suggested method is superior on its sensitivity, simplicity and low cost. Although, chromatographic methods like HPLC (with UV, fluorescence, or mass spectrometry detection) and GC are effective, they require extensive clean-up, making them labor-intensive and costly. The reported TLC method was tedious, involving sample extraction with ether under alkaline conditions, followed by a reaction before separation. While spectrophotometric methods are simpler, they lack sufficient sensitivity. Additionally, a conductometric method is noted for being non-selective.

## Electronic supplementary material

Below is the link to the electronic supplementary material.

Supplementary Material 1

## Data Availability

No datasets were generated or analysed during the current study.

## Abbreviations

HTM-HCl: Heptaminol hydrochloride
DNS-Cl: Dansyl chloride
RFI: Relative Fluorescence Intensity
DCM: Dichloromethane
LOD: Limit of Detection
LOQ: Limit of Quantitation

## References

[1] Bahloul M, Chaari A, Mbarek MN, Ben, Kallel H, Bouaziz M. Use of heptaminol hydrochloride for catecholamine weaning in septic shock. Am J Ther. 2012;19:8–17. 10.1097/MJT.0b013e3181e9b630 20720484

[2] Garrett J, Osswald W, Moreira MG. Mechanism of Cardiovascular actions of Heptanolamines. Br J Pharmacol Chemother. 1962;18:49–60. 13897064 10.1111/j.1476-5381.1962.tb01149.x PMC1482155

[3] Garrett J. Pharmacodynamic study of 6-amino-2-methyl-2-heptanol hydrochloride (heptaminol hydrochloride or RP 2831). I. Action of heptaminol hydrochloride on the cardiovascular and central nervous systems. Arch Int Pharmacodyn Ther. 1954;100:17–34. 14350831

[4] Pourrias B. Heptaminol chlorhydrate: new data. In: Annales Pharmaceutiques Francaises. 1991. pp. 127–38. 1929117

[5] The European Pharmacopeia. 8th ed., Council of Europe, strasburg, vol.II., 2014.

[6] Cociglio M, Sauvaire D. Liquid chromatographic assay of heptaminol in serum and its oral pharmacokinetics in the dog. J Chromatogr Biomedical Appl. 1984;307:351–9. 10.1016/s0378-4347(00)84106-7 6736182

[7] Sun YX, Zhao LM, He XJ, Qiu F. Determination of heptaminol in human plasma and urine by high-performance liquid chromatography. Chin Pharm J. 2008;43:179–86. 10.1016/s0378-4347(00)84421-7 6874820

[8] Nicolas A, Leroy P, Moreau A, Mirjolet M. Determination of heptaminol in pharmaceutical preparations by high-performance liquid chromatography. J Chromatogr A. 1982;244:148–52.

[9] Nováková L, Rentsch M, Grand-Guillaume Perrenoud A, Nicoli R, Saugy M, Veuthey JL, et al. Ultra high performance supercritical fluid chromatography coupled with tandem mass spectrometry for screening of doping agents. II: analysis of biological samples. Anal Chim Acta. 2015;853:647–59. 25467514 10.1016/j.aca.2014.10.007

[10] Badoud F, Grata E, Perrenoud L, Avois L, Saugy M, Rudaz S, et al. Fast analysis of doping agents in urine by ultra-high-pressure liquid chromatography-quadrupole time-of-flight mass spectrometry. I. Screening analysis. J Chromatogr A. 2009;1216:4423–33. 19342059 10.1016/j.chroma.2009.03.033

[11] Sardela VF, Sardela PDO, Deventer K, Araujo ALD, Cavalcante KM, Padilha MC, et al. Identification of sympathomimetic alkylamine agents in urine using liquid chromatography-mass spectrometry and comparison of derivatization methods for confirmation analyses by gas chromatography-mass spectrometry. J Chromatogr A. 2013;1298:76–85. 23746644 10.1016/j.chroma.2013.05.016

[12] Hsu KF, Chien KY, Chang-Chien GP, Lin SF, Hsu PH, Hsu MC. Liquid chromatography-tandem mass spectrometry screening method for the simultaneous detection of stimulants and diuretics in urine. J Anal Toxicol. 2011;35:665–74. 22080905 10.1093/anatox/35.9.665

[13] Ying LT, Liu CH, Kuo FH, Shieh MH. Solid-phase column chromatographic and gas chromatographic-mass spectrometric determination of heptaminol in human urine and related pharmacokinetic profiles. J Anal Toxicol. 2006;30:365–9. 16872566 10.1093/jat/30.6.365

[14] Rabiant J, Sergant M, Gaudin AF. Heptaminol determination by gas chromatography: application to the study of urinary excretion. In: Annales Pharmaceutiques Francaises. 1971. pp. 331–6. 5124763

[15] Kueh AJ, Marriott PJ, Wynne PM, Vine JH. Application of comprehensive two-dimensional gas chromatography to drugs analysis in doping control. J Chromatogr A. 2003;1000:109–24. 12877168 10.1016/s0021-9673(02)01998-2

[16] Morros A, Borja L, Segura J. Determination of heptaminol in plasma by thin-layer chromatography and in situ fluorimetry. J Pharm Biomed Anal. 1985;3:149–56. 16867697 10.1016/0731-7085(85)80018-2

[17] Varu HL, Kapuriya NP, Bhalodia JJ, Patel RB, Bapodra AH, Ambasana MA. An Expeditious Spectrophotometric Estimation of Memantine Hydrochloride by Facile Derivatization using N, N-Dimethyl aniline. J Anal Chem. 2022;77:1433–9.

[18] El Walily AFM, El-Yazbi FA, Belal SF, Abdel-Razak O. Spectrophotometric and spectrofluorometric determination of heptaminol and mexiletine in their dosage forms. Anal Lett. 1997;30:2029–43.

[19] Belal SF, Haggag RS, Shaalan RAA. The use of an aromatic substitution reaction in the spectrophotometric determination of selected amino or thiol containing drugs. J Food Drug Anal. 2008;16:26–33.

[20] Ayad M, Hosny MM, Elabassy M, Belal OM. Conductometric determination of Betahistine dihydrochloride and Heptaminol hydrochloride using silver nitrate. Zagazig J Pharm Sci. 2020;0:0–0.

[21] El-Adl SM. A new sensitive fluorimetric method for the determination of heptaminol and mexiletine in pharmaceuticals. Sci Pharm. 2002;70:57–65.

[22] Halim ME, Omar MA, Nagy DM. Condensation of heptaminol hydrochloride for its spectrofluorimetric determination in pure form and tablets: application in human plasma. Luminescence. 2020;35:821–6. 31994292 10.1002/bio.3787

[23] Omar MA, Nagy DM, Halim ME. Fluorescamine-based fluorophore for spectrofluorimetric determination of heptaminol in human plasma; application to spiked human plasma. Spectrochim Acta - Part Mol Biomol Spectrosc. 2020;227:117711. 10.1016/j.saa.2019.117711 31690484

[24] Derayea SM, Khashaba PY, Abdelgaleel M, Nagy DM. Orthophthaldehyde as a fluorescent probe for the feasible and sensitive assay of heptaminol: application to dosage forms and real human plasma. Luminescence. 2022;37:230–7. 34791769 10.1002/bio.4164

[25] Omar MA, Nagy DM, Halim ME. Utility of ninhydrin reagent for spectrofluorimetric determination of heptaminol in human plasma. Luminescence. 2018;33:1107–12. 29968975 10.1002/bio.3516

[26] Joshi RJ, Varu HL, Bhalodia JJ, Ambasana MA, Bapodra AH, Kapuriya NP. Highly selective fluorescence sensor based on azido pyrazole-chalcone conjugates for rapid detection of iodide ion. Results Chem. 2024;7:101409.

[27] Ulu ST. Sensitive spectrofluorimetric determination of tizanidine in pharmaceutical preparations, human plasma and urine through derivatization with dansyl chloride. Luminescence. 2012;27:426–30. 23044773 10.1002/bio.1367

[28] Karasakal A, Ulu ST. Sensitive spectrofluorimetric determination of alfuzosin in pharmaceutical preparations and human urine using dansyl chloride. J Anal Chem. 2015;70:708–11.

[29] Önal C, Kepekçi Tekkeli \cSerife Evrim. Spectrofluorimetric analysis of ticagrelor in pharmaceutical formulations and spiked human plasma using 1-dimethylaminonaphthalene-5-sulphonyl chloride reagent. J Res Pharm. 2020;24.

[30] Derayea SM, Abdelgaleel M, Nagi DM, Oraby M, Khashaba PY. Utility of dansyl chloride for the establishment of a sensitive spectrofluorimetric approach for estimating midodrine hydrochloride: application to content uniformity testing. RSC Adv. 2023;13:33453–8. 38025857 10.1039/d3ra06268f PMC10644863

[31] Hussein OG, Ahmed DA, Abdelkawy M, Rezk MR, Rostom Y. A novel green spectrofluorimetric method for simultaneous determination of antazoline and tetryzoline in their ophthalmic formulation. Luminescence. 2024;39:e4728. 38516711 10.1002/bio.4728

[32] Rostom Y, Hussein OG, Abdelkawy M, Rezk MR, Ahmed DA. A Novel Spectrofluorimetric determination of Antazoline and Xylometazoline in their Ophthalmic Formulation; Green Approach and evaluation. J Fluoresc. 2024;:1–14. 10.1007/s10895-024-03585-0 38319520

[33] Guideline ICHHT. Others. Validation of analytical procedures: text and methodology. Q2. 2005;1:5.

