# Supplementary material for: Application of nucleophilic substitution reaction for sensitive determination of heptaminol hydrochloride in pharmaceuticals
Source: BMC Chem. 2024 Nov 7;18(1):218. doi: 10.1186/s13065-024-01327-8 (PMC11545629; doi:10.1186/s13065-024-01327-8)
Supplement: Supplementary file 1 — Supplementary Material 1 [file 13065_2024_1327_MOESM1_ESM.docx]

**Supplementary Table 1: Glimpses of reported spectrophotometric and conductometric methods.**

| No. | Method | Conditions | Linear range (μg. ml^-1^) | LOD  (μg ml^-1^) | Ref. |
| --- | --- | --- | --- | --- | --- |
| 1 | Acetyl acetone/  Formaldehyde | Boiling W.B/10 minutes | 15-30 | 0.4 | [17] |
| 2 | 2,4-dinitrofluorobenzene (DNFB) | Warming to 40^0^C/ 40 minutes | 5-25 | 1.45 | [18] |
| 3 | Precipitation of chloride ions present in cited drug with silver ions, yielding silver chloride. | the conductance of the solution is measured as a function of the volume of titrant | 2000-13000 |  | [19] |

**Supplementary table 2: Analysis of the developed approach against the reported spectrofluorimetric methods.**

| No. | Reagent | λex/λem (nm) | Conditions | Linear range (μg. ml^-1^) | LOD  (μg ml^-1^) | Ref. |
| --- | --- | --- | --- | --- | --- | --- |
| 1 | Acetyl acetone/  Formaldehyde | 412/480 | Boiling W.B/10 minutes | 0.2–0.8 | 0.15 | [17] |
| 2 | DAS | 381/441 | Room temp./ Extraction to CHCl_3_/10 minutes | 0.016–0.144 | 0.003 | [20] |
| 3 | Ethyl acetoacetate/  Formaldehyde | 350/416 | Boiling W.B/30 minutes | 0.2-2.0 | 0.06 | [21] |
| 4 | Fluorescamine | 393/471 | 10 min/room temp. | 0.075–0.85 | 0.023 | [22] |
| 5 | Ortho-phthalaldehyde/ 2-mercapto-ethanol | 334/451 | 20 min./room temp | 0.005 – 0.1 | 0.001 | [23] |
| 6 | Ninhydrin/  Phenylacetaldehyde | 390/464 | 12 minutes/75^0^C | 0.5-6.0 | 0.14 | [24] |
| 7 | DNS-Cl | 345/490 | 25 minutes/ room temp. | 0.03-2.0 | 0.016 | This work |
